# Personalized Management of Fatigue in Individuals With Myalgic Encephalomyelitis/Chronic Fatigue Syndrome and Long COVID Using a Smart Digital mHealth Solution: Protocol for a Participatory Design Approach

**DOI:** 10.2196/50157

**Published:** 2024-04-12

**Authors:** Enrique Dorronzoro-Zubiete, Jesús Castro-Marrero, Jorge Ropero, José Luis Sevillano-Ramos, María Dolores Hernández, Ramon Sanmartin Sentañes, Jose Alegre-Martin, Patricia Launois-Obregón, Isabel Martin-Garrido, Asuncion Luque Budia, Juan R Lacalle-Remigio, Luis Béjar Prado, Octavio Rivera Romero

**Affiliations:** 1 Electronic Technology Department Universidad de Sevilla Sevilla Spain; 2 Research Unit in ME/CFS and Long COVID Division of Rheumatology Vall d'Hebron Research Institute, Universitat Autònoma de Barcelona Barcelona Spain; 3 Architecture and Technology Department Universidad de Sevilla Sevilla Spain; 4 Division of Rheumatology Vall d'Hebron University Hospital Universitat Autònoma de Barcelona Barcelona Spain; 5 Physical Medicine and Rehabilitation Department Vall d'Hebron University Hospital Barcelona Spain; 6 Department of Medicine Universitat Autònoma de Barcelona Barcelona Spain; 7 Unidad de Enfermedades Autoinmunes y Minoritarias, Servicio de Medicina Interna Virgen del Rocio University Hospital Sevilla Spain; 8 Salud Mental, Unidad de Gestión Clínica Virgen del Rocio University Hospital Sevilla Spain; 9 Preventive Medicine and Public Health Department Universidad de Sevilla Sevilla Spain

**Keywords:** acceptability, myalgic encephalomyelitis/chronic fatigue syndrome, long COVID, mHealth, fatigue, physical activity, lifestyle health, personalized self-management, user-centered design

## Abstract

**Background:**

Fatigue is the most common symptom in myalgic encephalomyelitis/chronic fatigue syndrome (ME/CFS) and long COVID, impacting patients’ quality of life; however, there is currently a lack of evidence-based context-aware tools for fatigue self-management in these populations.

**Objective:**

This study aimed to (1) address fatigue in ME/CFS and long COVID through the development of digital mobile health solutions for self-management, (2) predict perceived fatigue severity using real-time data, and (3) assess the feasibility and potential benefits of personalized digital mobile health solutions.

**Methods:**

The MyFatigue project adopts a patient-centered approach within the participatory health informatics domain. Patient representatives will be actively involved in decision-making processes. This study combines inductive and deductive research approaches, using qualitative studies to generate new knowledge and quantitative methods to test hypotheses regarding the relationship between factors like physical activity, sleep behaviors, and perceived fatigue in ME/CFS and long COVID. Co-design methods will be used to develop a personalized digital solution for fatigue self-management based on the generated knowledge. Finally, a pilot study will evaluate the feasibility, acceptance, and potential benefits of the digital health solution.

**Results:**

The MyFatigue project opened to enrollment in November 2023. Initial results are expected to be published by the end of 2024.

**Conclusions:**

This study protocol holds the potential to expand understanding, create personalized self-management approaches, engage stakeholders, and ultimately improve the well-being of individuals with ME/CFS and long COVID.

**International Registered Report Identifier (IRRID):**

PRR1-10.2196/50157

## Introduction

### Overview

Fatigue is recognized as one of the most common symptoms in individuals with chronic postviral conditions. It is defined as the early onset of tiredness after starting an activity, a feeling of exhaustion or difficulty in carrying out a physical (physical fatigue) or cognitive (cognitive fatigue) task, which is not recovered after a rest [1]. It is relevant to highlight several issues included in this definition. First, fatigue is a consequence of carrying out an activity, also referred to in the scientific literature as “postexertional malaise.” Furthermore, patients experience two different types of fatigue—physical and cognitive. Finally, fatigue is essentially a subjective condition, encompassing feelings or difficulties. Thus, it is linked to perceived fatigue, which is defined as the subjective lack of physical and/or mental energy that is perceived by the individual or caregiver to interfere with usual and desired activities [1]. Myalgic encephalomyelitis, also known as chronic fatigue syndrome (ME/CFS) and post–COVID-19 syndrome or postacute sequelae of SARS-CoV-2 infection (PASC), also known as long COVID, are multisystem postviral conditions where chronic fatigue is present as the most common disabling symptom. ME/CFS is a complex, multisystem, and profoundly debilitating neuroimmune condition. Although individuals with ME/CFS experience several symptoms, fatigue is the most disabling one [1].

Some symptoms associated with the SARS-CoV-2 infection may persist for a significant period. Long COVID or PASC is an umbrella term for the wide range of health consequences that are present 4 or more weeks after SARS-CoV-2 infection. Most cases infected with SARS-CoV-2 are asymptomatic or have mild acute symptoms with low rates of hospitalization and death. However, some of them, including those with mild or asymptomatic infection, develop postacute manifestations following SARS-CoV-2 infection. There are many symptoms presented in PASC, such as sleep difficulties and anxiety or depression [2], with fatigue being the most frequently reported.

The extent of fatigue in patients’ lives is huge, affecting their physical, cognitive, and socioeconomic conditions [3]. It affects several aspects of life, including physical and cognitive activity, decreasing adherence to treatments and recommendations, and affecting the ability to function or work. In addition, it is normally misunderstood by the social environment, causing isolation and frustration. Therefore, fatigue strongly affects patients’ well-being and significantly reduces their quality of life.

The effective fatigue management of ME/CFS and long COVID would minimize the consequences of the disease, significantly impacting patients’ and caregivers’ quality of life. The clinical evolution of ME/CFS and long COVID is still unpredictable. Recommendations and strategies should consider the individual’s physical and cognitive capabilities and other factors, such as daily perceived fatigue fluctuations, meteorological condition, level of physical activity, caffeine and alcohol consumption, sleep quality, mood, stress, and treatments. Many of those factors present a wide daily variability, and therefore, personalization must be driven by real-time data collected in a patient’s real-life setting. An effective personalization strategy may help to increase compliance with management recommendations and strategies.

Digital health allows the implementation of interesting functionalities for the management of long-term conditions, such as tracking activities, remote monitoring of an individual’s condition, real-time feedback, just-in-time recommendations, communication, educational content, and reminders. Digital health solutions have proven to be effective in the management of some chronic diseases, such as diabetes and cancer. Therefore, they may be effective as a self-management tool for individuals with ME/CFS or long COVID. Several recent studies have explored the effectiveness of internet-based cognitive behavioral therapies in fatigue management among individuals with ME/CFS [4,5]. Despite the potential benefits of digital health solutions for the self-management of ME/CFS and long COVID, there are no evidence-based, personalized, and context-aware solutions designed specifically for supporting patients with ME/CFS or long COVID in fatigue self-management.

### Current Status

Despite the promising benefits of using digital health for the self-management of chronic diseases, there is a lack of studies exploring the specific needs and preferences of individuals with ME/CFS or long COVID haulers regarding digital fatigue self-management. Only 1 study examined how adolescents use the internet to cope with ME/CFS [6]. Moreover, there is a lack of evidence on how digital health may benefit fatigue self-management in this group of patients.

Remote monitoring in real-time is a relevant functionality to personalize fatigue self-management in ME/CFS and long COVID. Several studies that remotely monitor the health conditions of patients with ME/CFS in real-time using mobile health (mHealth) devices have been recently published. Worm-Smeitink et al [5] conducted an ecological momentary assessment (EMA) study aimed to explore the associations between cognitions, physical and cognitive activity, social behaviors and their effects, as well as fatigue in ME/CFS. King et al [7] used an accelerometer device to objectively measure the physical activity of patients with ME/CFS, intending to classify them into different categories. Palombo et al [8] used a commercial inertial measurement unit to develop a sensor-based method to measure an indicator of ME/CFS disease severity. Josev et al [9] used an actigraphy device to measure the sleep quality of the pediatric ME/CFS population. Russell et al [10] examined the relationship between subjective and actigraphy-defined sleep and next-day fatigue in ME/CFS. However, to our knowledge, there are no studies examining the impact of a combination of contextual factors, physical activity level, sleep quality, and mood on the fatigue severity in ME/CFS and long COVID. Moreover, there are no studies focused on analyzing the similarities and differences in fatigue between ME/CFS and long COVID. Analyzing these contextual data and their relationships with perceived fatigue severity using artificial intelligence (AI) models may enable the definition of successful data-driven personalization strategies for fatigue self-management in ME/CFS and long COVID, leading to an increase in patients’ adoption and adherence.

Finally, AI models developed in ME/CFS and long COVID are focused on diagnosis, biomarkers, differentiating from other diseases, prevalence estimation, and association with anxiety or depression symptoms [11,12]. Therefore, there are no AI models to estimate fatigue severity based on contextual and behavioral factors in ME/CFS and long COVID.

### Hypothesis

Although the use of digital solutions to self-manage fatigue among people with chronic conditions, such as multiple sclerosis, has been previously studied, the wide range of potential symptoms and their unpredictability and variability in ME/CFS and long COVID define a completely different scenario, posing new challenges to be solved. In addition, the similarities and differences between fatigue in ME/CFS and long COVID are still unknown, and there is a need for new research aimed at discovering them and translating them into actionable recommendations to manage fatigue in long COVID. Defining effective fatigue self-management in ME/CFS and long COVID is crucial to increasing patients’ adherence to treatment, reducing mental health problems, and improving individual’s well-being and quality of life. The MyFatigue project will progress beyond the state of the art on ME/CFS and long COVID fatigue self-management, providing new insights and actionable recommendations to fill in the gap.

We hypothesize that the use of a personalized and context-aware digital health solution supporting individuals with ME/CFS or long COVID in fatigue self-management increases their self-efficacy, reduces the risk of having mental health problems, and improves their quality of life. We also hypothesize that fatigue in ME/CFS and long COVID presents similar patterns regarding contextual factors.

The MyFatigue project will generate new knowledge on the relevance and relationships of factors impacting perceived fatigue severity in ME/CFS and long COVID, analyzing similarities and differences between both study populations. MyFatigue will also explore the specific needs and preferences of individuals with ME/CFS and long COVID haulers for digital health solutions, supporting fatigue self-management. Based on this knowledge, a personalized and context-aware solution supporting patients in their fatigue self-management will be co-designed, and its acceptance and potential benefits will be assessed.

### Aims and Research Questions

The general aim of the MyFatigue project is to contribute to the understanding of fatigue in ME/CFS and long COVID, a modern medical challenge still unresolved, and to find opportunities for designing digital health solutions and supporting self-management to improve individuals’ quality of life. The specific aims of this study are as follows:

1. To identify attitudes of patients with ME/CFS and long COVID haulers toward the use of digital health solutions, supporting them in fatigue self-management and their specific needs and preferences
2. To determine the current digital health solutions being used by individuals with ME/CFS and long COVID haulers in their self-management
3. To analyze the relevance of contextual factors and behaviors in the perceived fatigue severity, their daily fluctuations, and their relationships
4. To identify similarities and differences in fatigue symptoms between ME/CFS and long COVID
5. To define patient clusters based on fatigue patterns and to identify relevant factors impacting each cluster
6. To predict the perceived fatigue severity based on real-time data on contextual factors, behaviors, and individuals’ conditions collected in real-life settings
7. To determine the feasibility, acceptance, and potential benefits of the use of a personalized and context-aware digital health solution for fatigue self-management in ME/CFS and long COVID

### Participants and Methods

#### Ethical Considerations

Ethical approval will be sought before beginning the recruitment of participants. Written informed consent will be sought from all study participants before the initiation of participant-related study activities, such as the implementation and evaluation of the personalized digital mHealth-solution–based management intervention.

#### Participant Recruitment and Study Design

Health care institutions, such as Hospital Universitario Virgen del Rocío and Hospital Universitari Vall d’Hebron, are involved in the MyFatigue project. The proposed studies will be conducted in both institutions. Individuals with ME/CFS and long COVID haulers will be invited to participate in these studies. For all the proposed studies, a sample of individuals with ME/CFS and another sample of individuals with long COVID haulers will be recruited in each hospital for each study. In the proposed study, to reach specific aims 3 and 4, a supplementary sample of control healthy individuals, exhibiting similar sedentary behaviors to those with ME/CFS and long COVID, will be recruited in each site.

#### Eligibility Criteria

Potential participants should have been diagnosed with ME/CFS (based on the 1994 Fukuda case definition) or long COVID (based on the 2021 World Health Organization clinical case definition), be older than 18 years at the time of recruiting, have none to moderate physical disability, and have communication ability in Spanish. Several factors, such as age and gender, will be considered to ensure a rich sample for analysis. Project objectives, risks, benefits, and their rights as research participants will be explained to potential participants. An information sheet will be provided to the participants, and they will sign an informed consent, indicating that they will be able to withdraw from the study at any time**.**

**Figure 1:**
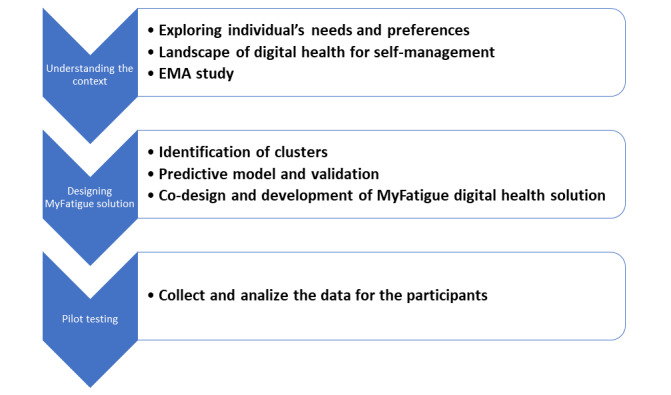
MyFatigue work plan. EMA: ecological momentary assessment.

The work plan and tasks for the MyFatigue project are presented in Figure 1.

### Understanding the Context

#### Exploring Individuals’ Needs and Preferences

A mixed methods study design using qualitative and quantitative methods will be conducted to reach the first objective. Individual semistructured interviews will be carried out with each sample of participants. Once participants sign the informed consent, a face-to-face semistructured interview will be conducted, with a remote interview when an in-person meeting is not possible. An experienced interviewer will lead the interview following the script defined in the protocol. Initially, the defined questions will be inquired during the session. Interesting topics not initially included will be identified and added iteratively to be used during the following sessions. In the same way, irrelevant questions will be removed as the study progresses. All sessions will be recorded, digitally transcribed, and anonymized. Transcripts will be coded, and thematic analysis will be performed in an iterative process. Each participant’s comment will be categorized as barriers or facilitators. Further refinement will take place by merging and removing redundant themes until consensus is reached.

Once data analysis ends, the findings will be discussed among all team members participating in the task. A deeper analysis of similarities and differences between findings found in ME/CFS and long COVID will be performed. We will also compare these findings with the findings in the study that is being conducted on multiple sclerosis. Once consensus is reached, we will validate the findings involving some of the participants in the interviews. We will apply a gender perspective in recruitment, data analysis, interpretation of findings, and validation of results.

#### Landscape of Digital Health for Self-Management

A patient survey will be designed by a group of experts involving health care professionals and computer engineers. The survey will aim to analyze the technological solutions that individuals with ME/CFS and long COVID haulers are currently using to self-manage their conditions, especially fatigue. The questionnaire will include sociodemographic variables, disease-related data, and respondents’ general opinions on digital health solutions. It will also include questions about digital literacy and access, the advantages and disadvantages of using technology for fatigue management, perceived barriers in the use of these devices, personal data management, and general functionalities.

Anonymized data will be collected, complying with the current regulations. Web-based questionnaires will be tested on a group of individuals with ME/CFS or long COVID and on professionals to optimize format and wording and to assess their usability before their large-scale administration. Discrepancies among reviewers’ comments will be solved by consensus, and a final version of the questionnaire will be developed including the reviewers’ suggestions. Participation will be voluntary and anonymous, and duplicate responses will be avoided. Data collected through the questionnaires will be analyzed, and descriptive statistics will be calculated using frequencies and cross-tabulations by key demographic and disease-related variables. Age and gender will be taken into consideration during the analysis. We expect the participation of at least 20 individuals per group (ME/CFS and long COVID).

### EMA Study

The proposed quantitative study is based on EMA and continuous data collection using wearable devices (eg, Actigraphy GT3X). The research team has developed a mobile app that enables the remote monitoring of individuals’ conditions through a questionnaire. The MyFatigue app will be developed to perform the EMA study. The app will allow participants to self-report fatigue severity during periods when they are not wearing the device required to distinguish low levels of activity from noncompliance, among other factors. The proposed study will begin with one-to-one introductory sessions during which the app and wearable device will be provided. They will be instructed about its use, and their demographic and relevant clinical data will be collected. Finally, participants will complete a set of standard questionnaires to define a baseline. The questionnaires to be filled include the Chalder Fatigue Questionnaire (CFQ), the Modified Fatigue Impact Scale (MFIS-5), the eHealth scale, the Satisfaction with Life Scale (SWLS), The Pittsburgh Sleep Quality Index (PSQI), and the Hospital Anxiety and Depression Scale (HADS). Participants will wear the device and self-report using the app for 14 consecutive days. They will be asked to carry out their regular daytime activities and to keep their usual sleep or wake schedules. The study will be conducted in 4 waves, collecting data in different seasons and avoiding insufficient data due to device failure or losses. We estimate that 120 individuals will participate in each wave. Participants will report the perceived fatigue severity using this app, by responding to a single-item questionnaire. At least, 3 self-reports per day (morning, afternoon, and evening) will be carried out, prompted by the app. Participants will be able to set reminders, postpone them, and silence them, if necessary. Moreover, they can carry out additional self-reports if they feel fatigued. Additionally, the app will collect data on potential factors like temperature, meteorological conditions, mental activity level, and mood.

Physical activity and sleep data will be continuously recorded using an actigraphy device worn on the dominant wrist. The device will be set to record in 1-minute epochs using zero crossing mode. Actigraphy data will be analyzed using a specific software, such as ActiLife. Raw data will be converted to counts, which will be analyzed to identify and calculate different physical activity variables. The intensity and absence of activity will be classified according to Fjeldstad et al [13] and based on the following cut-off points: sedentary (<1.5 metabolic equivalent of task [MET]: 0-199 counts per minute [CPs]); light (1.5-2.99 MET: 200-1952 CPs); moderate (3-5.99 MET: 1953-5724 CPs); hard (6.0-8.99 MET: 5725-9498 CPs); and very hard (>9.0 MET: > 9498 CPs). Other data, such as light conditions, environmental temperature, systolic blood pressure and diastolic blood pressure, heart rate, and heart rate variability could be collected if selected devices and budget allow it.

Descriptive statistics will be calculated, and Pearson correlation analysis will be performed among physical activity, sleep variables, and fatigue severity. Similarities and differences between ME/CFS and long COVID will be identified. Once the study period ends, a new in-person session will be conducted in which participants will return the device and fill out a questionnaire to identify any barriers they found in the use of the digital health solutions used in the EMA study. As a result of this task, a report on the statistical analysis findings will be delivered.

### Designing the MyFatigue Digital mHealth Solution

#### Identification of Clusters

As a first step, we will identify and validate clusters in data collected based on patients who present similar fatigue patterns, considering the contextual data collected. The clusters will be identified using unsupervised learning algorithms and validated with 2 different methodologies. A validation based on a graphical representation will be conducted, and some groups of clusters could be discarded. The remaining groups of clusters will be validated and selected using the following process: first, the research team members with experience in AI will review them and assess their representativeness of the collected data; then, the groups that better represent the collected data and clusters that are easier to understand will be selected by consensus; next, a definition of persona (a representation of a group of patients) for each cluster will be developed; then, a validation through a participatory workshop involving health care professionals of the research team will be conducted.

#### Predictive Modelling and Validation

To estimate the relationships between perceived fatigue severity and the remaining variables included in the data set and to estimate the fatigue severity, an analysis will be performed using blocks based on the definition of temporal windows. We will develop 2 interpretable models for fatigue severity forecasting: one using decision tree algorithms and the other using autoregressive integrated moving averages. The models will be validated through cross-validation, and their performance will be compared.

#### Co-Design and Development of the MyFatigue Digital Health Solution

The proposed solution will be developed following user-centered design (UCD), using a co-design workshop that involves patients in the design process. This involvement will present challenges because of the combination of symptoms that patients may present. Cognitive fatigue could pose a great challenge because fatigue severity may increase rapidly when individuals engage in dialogues with multiple participants. We will define a workshop plan that controls the number of activities to be done, plans frequent breaks, and limits the number of participants in each session. Once the session ends, we will send a questionnaire to participants in which we will ask them to assess the barriers and advantages that they found during the session. Based on UCD, the collected data will be analyzed to identify relevant aspects and challenges in involving individuals with fatigue and other symptoms in the design process and steps to successfully address them. Following our research through a design method based on UCD, we will design and develop a prototype of the MyFatigue digital health solution. This prototype will be evaluated by experts to assess its usability, accessibility, and appropriateness for patients with ME/CFS and long COVID haulers.

### Pilot Study

Through an initial in-person session, a member of the research team will provide the required device to participants and support them in the installation and configuration of the MyFatigue digital health solution. The researcher will also instruct participants on how they must process during their participation in the pilot study. A sheet summarizing those instructions and a tutorial on the MyFatigue digital health solution will be provided to participants. The pilot study will last 4 months. User logs for each participant will be saved in the MyFatigue database. After this period a new in-person session will be conducted in which a team member will ask participants to fill in an acceptance questionnaire and several standard questionnaires to assess the level of anxiety or depression, health-related quality of life, and the frequency or severity of fatigue. The acceptance questionnaire will be designed and validated by experts based on the Technology Acceptance Model. The collected data will be analyzed for each group of participants, paying special attention to relevant issues such as age and gender. Similarities and differences between both diseases will be explored.

## Results

The MyFatigue project started in September 2022. Intensive administrative tasks alongside technological advancements have been underway, including the development of the web server and mobile app, as well as comprehensive literature review to identify and select appropriate questionnaires and hardware (eg, actigraphy devices) to be used for the project. The project is expected to be concluded in 2025, and initial findings would likely be published by the end of 2025.

## Discussion

### Expected Outcomes

The MyFatigue project aims to make a significant scientific and technical impact in the field of ME/CFS and long COVID. It seeks to advance the understanding of fatigue by investigating the influence of contextual and behavioral factors on its severity and identifying similarities and differences between ME/CFS and long COVID symptoms. Additionally, the project aims to develop intelligent personalized fatigue self-management methodologies using digital health and patient-reported outcomes, leading to the creation of new personalized management strategies for these conditions.

The project also emphasizes the social and economic impact it intends to generate based on the theory of change, which is a methodology or criterion for planning, participation, adaptive management, and evaluation for promoting social change. By involving people with ME/CFS and long COVID as well as health care professionals in participatory research activities, the project aims to promote mental health and well-being, aligning with the Sustainable Development Goal target 3.4. Through training activities and dissemination efforts, the project aims to increase empowerment and self-efficacy among people with these conditions, potentially reducing the risk of mental health problems.

The MyFatigue project expects to have a positive impact on the quality of life of patients with ME/CFS and long COVID. The project will track impact indicators, such as the number of individuals who experience increased empowerment and self-efficacy, the number of students completing the Massive Open Online Course training, and the number of people who reduce their risk of mental health problems by 20% after participating in the project’s activities.

### Conclusions

The result of the described study has the potential to advance knowledge, develop personalized management strategies, involve stakeholders, and contribute to improving the well-being of individuals with ME/CFS and long COVID.

## Appendix

Multimedia Appendix 1 Peer-review report from the Spanish Ministry of Science and Innovation (Spanish).

Multimedia Appendix 2 Peer-review report from the Spanish Ministry of Science and Innovation (English).

## Abbreviations

AI: artificial intelligence
CFQ: Chalder Fatigue Questionnaire
CP: count per minute
EMA: ecological momentary assessment
HADS: Hospital Anxiety and Depression Scale
ME/CFS: myalgic encephalomyelitis/chronic fatigue syndrome
MET: metabolic equivalent of task
MFIS-5: Modified Fatigue Impact Scale
mHealth: mobile health
PASC: postacute sequelae of SARS-CoV-2
PSQI: Pittsburgh Sleep Quality Index
SWLS: Satisfaction with Life Scale
UCD: user-centered design

## Data Availability

Data sharing is not applicable to this manuscript, as no data sets were generated or analyzed during the development of this protocol. Once data collection begins, all investigators will have access to anonymized patient-level data.
