# Supplementary material for: Personalized Management of Fatigue in Individuals With Myalgic Encephalomyelitis/Chronic Fatigue Syndrome and Long COVID Using a Smart Digital mHealth Solution: Protocol for a Participatory Design Approach
Source: JMIR Res Protoc. 2024 Apr 12;13:e50157. doi: 10.2196/50157 (PMC11053387; doi:10.2196/50157)
Supplement: Multimedia Appendix 1 [file resprot_v13i1e50157_app1.pdf]

MINISTERIO
DE CIENCIA
E INNOVACIÓN

AGENCIA ESTATAL DE INVESTIGACIÓN

Proyectos de Generación de Conocimiento 2021<br>Modalidad: Investigación Orientada Tipo B	
**Referencia:**	PID2021-125528OB-I00
**Área:**	Tecnologías de la información y de las comunicaciones
**Subárea:**	Ciencias de la computación y tecnología informática
**Investigador/a principal**	RIVERA ROMERO, OCTAVIO
**Título:**	GESTION PERSONALIZADA EN EL MOMENTO JUSTO DE LA FATIGA EMPLEANDO UNA SOLUCION DIGITAL INTELIGENTE CONSCIENTE DEL CONTEXTO: APROXIMACION PARTICIPATIVA CENTRADA EN EL PACIENTE

PROYECTOS DE GENERACIÓN DE CONOCIMIENTO 2021. MODALIDAD: INVESTIGACIÓN ORIENTADA TIPO B

INFORME DE VALORACIÓN CIENTÍFICO TÉCNICA - COMISIÓN TECNICA

PARTE 1

CRITERIOS DE EVALUACIÓN

1.Calidad y viabilidad de la propuesta

UMBRAL 30

Puntuación de 0 a 40: 36.0

1.1. Calidad

Este proyecto presenta una propuesta sólida y consistente. La propuesta está bien justificada y queda clara su relevancia. La contribución de la propuesta a la generación de conocimiento del ámbito de la propuesta está bien argumentada. Los objetivos están definidos con claridad y precisión.

Puntuación 0 a 30: 27.0

1.2 Viabilidad

La viabilidad queda bien demostrada. Las actividades propuestas son adecuadas para alcanzar los objetivos planteados. La distribución de tareas entre los miembros del equipo de investigación es adecuada.

Puntuación 0 a 10: 9.0

2. Equipo de investigación

UMBRAL 20

El equipo de investigación posee la experiencia adecuada para conseguir los objetivos del proyecto. Los miembros del equipo acreditan su capacidad para desarrollar el proyecto.

Puntuación 0 a 30: 25.0

3. Impacto

UMBRAL 10

Puntuación de 0 a 30: 27.0

3.1. Impacto científico-técnico de los resultados esperados

El proyecto promete conseguir avances de conocimiento importantes y contribuciones significativas a la solución de necesidades de la prioridad temática seleccionada. El plan de comunicación y diseminación científica está bien definido.

Puntuación 0 a 20: 17.5

3.2. Impacto social y económico de los resultados

Los resultados del proyecto tienen impacto social muy claro. Se valora positivamente la colaboración con empresas que aportará al proyecto a conseguir el impacto socio-económico. Teniendo en cuenta las consideraciones anteriores, el carácter competitivo de la convocatoria y las disponibilidades presupuestarias no ha sido posible conceder toda la financiación solicitada.

Puntuación 0 a 10: 9.5

Valoración Global

Puntuaciónde 0 a 100: 88.0

PARTE 2

OTROS ASPECTOS A CONSIDERAR

a) Aspectos relacionados con zonas polares o campañas oceanográficas(Cumplimentar solo en los proyectos que proceda)

No aplica

b) Condiciones específicas para la ejecución de determinados proyectos (Cumplimentar solo en los proyectos con aspectos relacionados con las condiciones o implicaciones recogidas en el Anexo IV de la convocatoria).

No aplica

INFORME-EXPERTO 1

PARTE 1

CRITERIOS DE EVALUACIÓN

1.Calidad y viabilidad de la propuesta

1.1 Calidad

La propuesta está orientada claramente a la prioridad temática seleccionada: 1.- Salud. Pretende estudiar el impacto de la combinación de distintos factores en la fatiga severa en encefalomielitis miálgica/síndrome de fatiga crónica (ME/CFS) y COVID persistente, así como la similitud y diferencias con el uso de herramientas de Inteligencia Artificial (IA), lo que es una necesidad de la prioridad temática seleccionada. La propuesta justifica adecuadamente esta necesidad y su relevancia para el bienestar de los pacientes con fatiga severa. La hipótesis de partida está bien fundamentada, teniendo similitudes con estudios previos, lo que resta originalidad a la propuesta. Los objetivos específicos están claramente presentados, teniendo en cuenta desde el estudio del estado del arte actual, hasta el análisis de los beneficios del producto final. Son acordes al equipo de trabajo y a la duración establecida. Sin embargo, los objetivos relativos al avance científico y tecnológico en las herramientas de IA a utilizar son limitados. El carácter multidisciplinar es amplio con participantes de distintos campos, siendo los predominantes informática y medicina. Sin embargo, no están suficientemente balanceadas con respecto a las tareas programadas.

B (Muy Bueno)

1.2 Viabilidad

Se definen distintas actividades para lograr todos los objetivos propuestos, sin embargo, no está suficientemente justificado que sean suficientes para el fin que se persigue, pues se aprecia que los pacientes tienen que rellenar un gran número de cuestionarios, lo que muestran un riesgo importante. El número de variables a considerar de forma automática es limitado. Encontrar los pacientes suficientes para obtener la base de datos inicial, así como para la validación es una tarea complicada, que se tiene en cuenta en la propuesta como un riesgo de forma acertada, aunque el plan de mitigación es insuficiente para justificar su cumplimiento, debido principalmente al compromiso que deben adquirir los pacientes. La experiencia previa y los desarrollos actuales del Equipo avalan la consecución de las tareas como están previstas. La distribución en tareas es adecuada y cuentan con los recursos necesarios para llevar a cabo las actividades. No se justifica convenientemente en la propuesta la partida destinada a personal, tampoco el tiempo que tendrían que estar contratados.

C (Bueno)

2. Equipo de investigación

El IP tiene una experiencia previa suficiente para liderar la propuesta, aunque no tiene una gran experiencia en tareas de liderazgo de proyectos. El equipo de investigación, en general, tiene una trayectoria adecuada para desarrollar las tareas encomendadas, aunque algunos de los integrantes han disminuido su intensidad en producción científica en los últimos años. Existe una colaboración previa con investigadores extranjeros, algunos de los cuales se incluyen en el Equipo de Trabajo, aunque no se advierte una intensificación presencial de estas colaboraciones en la propuesta y presupuesto.

C (Bueno)

3. Impacto

3.1. Impacto científico-técnico

El producto final será determinante para la detección de la fatiga severa, por lo tanto, en este aspecto, contribuirá en una necesidad de la prioridad temática. Sin embargo, no está clara su utilidad más allá de dar un diagnóstico recomendado. En la determinación de variables representativas de las fatigas de ME/CFS y del COVID persistente, y de sus diferencias será un hito en la materia, no obstante, la consecución de este objetivo no está suficientemente justificado. Además, los avances científicos esperados en IA serán limitados. El carácter multidisciplinar de la propuesta propiciará un producto final más robusto y con mejores resultados. Se presenta un plan de difusión estándar basado en la publicación de artículos científicos en congresos y revistas, planteando indicadores específicos, aunque poco ambiciosos. El plan de transferencia se basa principalmente en el interés de varias empresas en los resultados del proyecto, lo cual es destacable, aunque limitado. El plan para el tratamiento de datos está muy bien enmarcado y diseñado.

D (Aceptable)

3.2. Impacto social y económico

El impacto social es claro a través de los ciudadanos con problemas de fatiga intensa. La propuesta presenta un adecuado plan de difusión de los resultados a la sociedad, que se basa esencialmente en la edición de una página web, el uso de las redes sociales y de notas de prensa. También considera indicadores para medir el impacto de las actividades programadas. Se considera la dimensión de género de forma adecuada. También se tienen en cuenta y son una parte fundamental para el proyecto los usuarios finales, aunque una participación masiva de estos no está garantizada.

C (Bueno)

Valoración Global

C=Bueno

PARTE 2

OTROS ASPECTOS A CONSIDERAR

a) Capacidad formativa (cumplimentar solo cuando se haya solicitado la inclusión del proyecto en la convocatoria de contratos predoctorales para la formación de doctores). En caso de No aplicar, indicar No aplica en el desplegable y en la caja de texto.

No aplica.

No aplica

b) Aspectos relacionados con zonas polares o campañas oceanográficas (cumplimentar solo en los proyectos que proceda)

No aplica.

c) Condiciones específicas para la ejecución de determinados proyectos (cumplimentar solo en los proyectos con aspectos relacionados con las condiciones o implicaciones recogidas en el Anexo IV de la convocatoria).

Justifica adecuadamente los procedimientos y protocolos que prevé aplicar.

INFORME-EXPERTO 2

PARTE 1

CRITERIOS DE EVALUACIÓN

1.Calidad y viabilidad de la propuesta

1.1 Calidad

El proyecto se adecua a la prioridad temática del PEICTI y se enmarca dentro del ámbito de la salud y el bienestar. La propuesta es clara e interesante y ataca un tipo de problemática con mucho potencial. La propuesta es relativamente novedosa y está bien justificada. Y la hipótesis de partida es original y novedosa. Los objetivos son claros y realistas y acordes con la duración del proyecto. La contribución de la propuesta generará resultados importantes en cuanto a generación de conocimiento. Se trata de una propuesta multidisciplinar con participación de investigadores de ámbitos diferentes.

B (Muy Bueno)

1.2 Viabilidad

Las actividades propuestas se ajustan a los objetivos propuestos y se sugiere una metodología correcta con una adecuada distribución de tareas e identificación de puntos críticos. Los resultados previos en cuanto a la publicación de resultados y participación en proyectos avalan la viabilidad de la propuesta. El equipo cuenta con recursos y potencial suficiente para la consecución de los objetivos del proyecto. El presupuesto presentado es razonable aunque con algunos aspectos poco justificados como el personal y los pagos por publicar en abierto.

B (Muy Bueno)

2. Equipo de investigación

El IP presenta un buen CV con contribuciones relevantes en diferentes aspectos aunque no ha liderado antes proyectos del plan nacional, aunque sí ha participado en proyectos relevantes para la presente propuesta. En general se considera que tiene méritos suficientes para liderar la propuesta. El equipo es claramente multidisciplinar con contribuciones relevantes y un historial reciente de publicaciones compartidas. La especialización de sus miembros es adecuada y la externalización de la investigación propuesta en 2 hospitales de diferentes ámbitos geográficos se considera positiva. Se aprecia un grado de internacionalización alto con relaciones interesantes con grupos e investigadores relevantes. La participación y distribución del equipo en los objetivos y tareas de proyecto es adecuada.

B (Muy Bueno)

3. Impacto

3.1. Impacto científico-técnico

La propuesta tiene un grado alto de novedad y por lo tanto de riesgo a la hora de valorar el impacto científico de los posibles resultados. Aún así, se considera interesante en este aspecto. Se prevé un avance significativo del conocimiento en los diferentes ámbitos. Aunque en la propuesta se aprecia un mayor detalle en los datos y en la definición de los problemas desde el punto de vista médico en relación a los problemas tecnológico-científicos. El plan de comunicación de resultados es adecuado y se propone un plan de internacionalización y valorización de resultados a través de relaciones con empresas. La gestión de datos generados por el proyecto es adecuada.

B (Muy Bueno)

3.2. Impacto social y económico

La propuesta implica un impacto social claro dado el objeto de estudio. El plan de difusión de resultados es adecuado y existe una dimensión de género naturalmente asociada a las patologías consideradas. En general se percibe un beneficio claro para la sociedad. El proyecto considera adecuadamente la participación de usuarios finales y ésta se gestiona adecuadamente.

A (Excepcional)

Valoración Global

A=Excepcional

PARTE 2

OTROS ASPECTOS A CONSIDERAR

a) Capacidad formativa (cumplimentar solo cuando se haya solicitado la inclusión del proyecto en la convocatoria de contratos predoctorales para la formación de doctores). En caso de No aplicar, indicar No aplica en el desplegable y en la caja de texto.

-

No aplica

b) Aspectos relacionados con zonas polares o campañas oceanográficas (cumplimentar solo en los proyectos que proceda)

-

c) Condiciones específicas para la ejecución de determinados proyectos (cumplimentar solo en los proyectos con aspectos relacionados con las condiciones o implicaciones recogidas en el Anexo IV de la convocatoria).

-

INFORME-EXPERTO 3

PARTE 1

CRITERIOS DE EVALUACIÓN

1.Calidad y viabilidad de la propuesta

1.1 Calidad

La propuesta presentada en este proyecto MyFatigue es la #Gestión personalizada en el momento justo de la fatiga empleando una solución digital inteligente consciente del contexto: Aproximación participativa centrada en el paciente# y se adecua perfectamente a la prioridad temática 1. Salud del PEICTI en el área temática TIC # INF y BME # DPT Herramientas diagnósticas, pronósticas y terapeúticas. La propuesta es novedosa y en cuanto a la aplicación a este síntoma que aparece asociado a las afecciones post-virales, como la Encefalomielitis Miálgica/Síndrome de Fatiga Crónica (EM/SFC) y el COVID persistente. Esta fátiga como detallan en la presentación es de gran relevancia dado el impacto en la salud del paciente. Los objetivos están muy bien definidos y están involucrados expertos tanto clínicos como técnicos asegurando el éxito del proyecto (informáticos, médicos, psicólogos). La aportación de este proyecto puede ser muy relevante dado el gran numero de afectados por la pandemia. El proyecto es ambicioso pero la experiencia previa de los investigadores y la inclusión en el equipo otros centros con los que ya se cuenta con experiencias previas y asociaciones de pacientes asegura su buen desarrollo. Por todo ello creo que el proyecto puede valorarse como excepcional

A (Excepcional)

1.2 Viabilidad

El producto final Myfatigue es un software médico que utiliza un hardware (activimetro de reloj de pulsera) que es producto sanitario del mercado (debe tener marcado CE como producto sanitario) y móviles / tablets / ordenadores convencionales para proporcionar información que se utiliza para tomar decisiones con fines terapéuticos. Este software médico resultado del proyecto tiene la consideración de producto sanitario según el reglamento (EU) 2017/745 (MDR) y se clasifica como clase IIa según la regla 11 del anexo VIII. Por ello precisa de la intervención de un Organismo Notificado para su evaluación de conformidad y para poder transferir el resultado de este proyecto a un cliente final y que sea viable deberíamos contemplar los requisitos reglamentarios. No parece contemplado el seguir normativa armonizada para el desarrollo del software tal como la EN 62304 y la EN 82304 y las asociadas a los ensayos de usabilidad EN 62366, la gestión de riesgos EN 14971 + EN 80002-1, el manual de uso EN ISO 201417, el sistema de calidad EN ISO 13485 y la aplicable a investigación clínica EN 14155, ... compilando todos estos en una documentación técnica siguiendo anexo II y III del reglamento, sin cuya evidencia de aplicación el resultado final no puede transferirse a una empresa para la obtención del marcado CE según MDR preceptivo para su comercialización. Se incluye la compra de los activímetros Actigraph GT3X que están autorizados por la FDA (k181077) para su uso en US pero que no cuentan con el, preceptivo para su uso en Europa, marcado CE con intervención de Organismo Notificado (indican que son clase I sin función de medición # dando en cambio medidas de ritmo cardiaco), se recomienda utilizar ya que se usa para medir un producto con marcado CE con ON (e.g. Respironics Actiwatch 2) Parece que no se han considerado los aspectos regulatorios de este proyecto o bien no han sido incorporados explicitamene en la solicitud por ello entendiendo que estos requisitos pueden incorporarse sin problemas por el equipo valoro el proyecto como A.

A (Excepcional)

2. Equipo de investigación

El IP1 no ha sido nunca IP principal pero cuenta con experiencia y liderazgo en otros proyectos y la participación de otros investigadores relevantes que aseguran la viabilidad de este proyecto. El CV de los miembros del proyecto es impresionante con relación a la temática y desarrollo propuesto por lo que su exito está asegurado. El proyecto en si se basa en la aplicación de experiencia previa en le proyecto MSF-PHIA app por lo que esta bien elaborado y justifido su desarrollo y salidas de publicaciones científicas del mismo. Por todo ello creo que el proyecto puede valorarse como excepcional

A (Excepcional)

3. Impacto

3.1. Impacto científico-técnico

La propuesta es novedosa y en cuanto a la aplicación a este síntoma que aparece asociado a las afecciones post-virales, como la Encefalomielitis Miálgica/Síndrome de Fatiga Crónica (EM/SFC) y el COVID persistente. Esta fátiga como detallan en la presentación es de gran relevancia dado el impacto en la salud del paciente, asegurando que los resultados serán novedosos y objeto de publicación en las revistas científicas y congresos. El plan de transferencia y valorazación no se desarrolla ya que posiblemente no se ha contemplado al ser

un objetivo a tres años vista y siendo un riesgo el no contemplar los requisitos reglamentarios. No obstante, creo que el impacto puede valorarse como excepcional

A (Excepcional)

3.2. Impacto social y económico

El plan de difusión / divulgación está muy desarrollado desde el punto de vista científico de los distintos colaboradores e IPs, la metodología contempla el tratamiento de genero y discapacidad adecuadamente. Los resultados incluyen un software médico incorporando IA que deben ser elaborados dentro de un sistema de calidad y recogiendo todos los requisitos reglamentarios para así ser directamente aplicables a su explotación posterior. Aún en el caso de pensar en un software no comercializable sino para uso propio del centro sanitario (in-house) este está contemplado en el nuevo reglamento MDR y se exigen los requisitos reglamentarios de cumplimiento de RGSF de anexo I aplicandose así toda la normativa indicada. El interés de seis empresas en los posibles resultados del proyecto demuestra la relevancia de este proyecto que sin duda será objeto de transferencia al mercado. Creo que el impacto puede valorarse como excepcional

A (Excepcional)

Valoración Global

A, Excepcional

PARTE 2

OTROS ASPECTOS A CONSIDERAR

a) Capacidad formativa (cumplimentar solo cuando se haya solicitado la inclusión del proyecto en la convocatoria de contratos predoctorales para la formación de doctores). En caso de No aplicar, indicar No aplica en el desplegable y en la caja de texto.

NA

No aplica

b) Aspectos relacionados con zonas polares o campañas oceanográficas (cumplimentar solo en los proyectos que proceda)

NA

c) Condiciones específicas para la ejecución de determinados proyectos (cumplimentar solo en los proyectos con aspectos relacionados con las condiciones o implicaciones recogidas en el Anexo IV de la convocatoria).

Con respecto a los aspectos éticos el proyecto detalla su aplicación en el estudio piloto, pero debe incorporarlo en todos los preliminares donde participan los pacientes y se indica que se solicita aprobación de los CEIm de los centros sanitarios, así parece establecerlo el uso de consentimientos informados. Al ser MyFatigue un programa informático con consideración de producto sanitario debe obtenerse además de la aprobación de los Comités Eticos, la aprobación de la AEMPS. Según el nuevo reglamento MDR deberá presentarse la solicitud en la base de datos europea EUDAMED (si esta operativa al realizar los ensayos).

INFORME-EXPERTO 4

PARTE 1

CRITERIOS DE EVALUACIÓN

1.Calidad y viabilidad de la propuesta

1.1 Calidad

La propuesta presentada es exhaustiva, pero, en ocasiones, la exposición es superficial. Es decir, algunos aspectos y/o secciones no han sido suficientemente desarrollados, concretados y/o justificados. Ejemplos de esta falta de concreción serían: (1) se establecen dos grupos de pacientes (más grupos controles), pero no se describen de forma detallada ni sus características principales, ni los criterios de inclusión y exclusión (Task 1.2, Task 3.1); (2) la metodología propuesta para la curación de los datos (Tasks 2.1, 2.2, SO5), basada principalmente en identificar clusters, se ve comprometida por una asunción simplista, que desdeña algunos factores contextuales. Es decir, la propuesta asume que la dimensión física del constructo de "fatiga", basada en la información (subjetiva y de actividad motora) proporcionada por un/una paciente, es independiente del tipo de actividad durante los 14 días, y trabaje en la construcción sería equivalente a trabajar en una oficina. Además, considerando siempre el aspecto metodológico, se describe un estudio longitudinal de 14 días (Task 1.5), pero la aproximación estadística obvia la relación temporal entre las variables. Por otra parte, no queda claro si en los 14 días de recogida de datos, se incluirán los días de adaptación (normalmente 3) que se suelen descartar cuando se trabaja con actigrafía. Finalmente, la decisión de posicionar el actígrafo en la muñeca dominante (en lugar de la solución más común y recomendada: muñeca no dominante) o la recogida de otras variables psicofisiológicas (tasa cardíaca, etc.) no se justifican de forma clara. Las hipótesis de investigación presentadas son muy genéricas y difícilmente refutables. Dada la experiencia ganada/derivada del proyecto "Understanding Daily Multiple Sclerosis related Fatigue: a Participatory Health Informatics Approach", en el que el mismo IP y algunos miembros del grupos de investigación y trabajo participan, no sería justificable alegar que es debido a que el proyecto tiene una natura exploratoria. Es más, la utilización de búsquedas preliminares (apartado 2.3) para motivar la importancia del proyecto no están justificadas en un equipo con experiencia en el tema. Dichas búsquedas usan ecuaciones de búsquedas claramente sesgadas (de idioma, de base de datos, o la inclusión de limitadores de búsqueda arbitrarios) para proporcionar escasos resultados y no presentan un estado del arte realista y completo.

C (Bueno)

1.2 Viabilidad

Aunque el equipo de investigación y de trabajo reflejan la naturaleza multi/interdisciplinar de la propuesta, el proyecto sigue una aproximación demasiado tecno-céntrica. Por ejemplo, se hace referencia al diseño basado en el usuario (UCD, task 2.4), pero el prototipo del MyFatigue será evaluado por expertos en usabilidad (en lugar de usuarios/as, que son los pacientes). Además, el desarrollo del sistema no sigue un proceso reiterativo diseño-test-rediseño típico del UCD. Así, la organización de los paquetes de trabajo no garantizaría el correcto desarrollo del sistema. O sea, los resultados del WP3 (testing), empezando sólo en los últimos meses del proyecto, no realimentarían al WP2. Finalmente, el equipo carece de expertos/as en factores humanos y experiencia del usurario que puedan liderar correctamente esta tarea. La presencia de expertos/as en psicología o intervención psicológica, que debería ser la parte más relevante del CATCH, es anecdótica en comparación con los otros perfiles. Por otra parte, no queda claro si la MyFatigue App-PRO ha sido desarrollada para entornos Android o IOS (o ambos). En el caso que al App fuera compatible sólo con un entorno específico, no se presenta una estrategia de contingencia (por ejemplo, proporcionar un dispositivo móvil compatible con la App). Considerando la vida útil del proyecto y el presupuesto considerado, el desarrollo de 7 objetivos específicos así como el reclutamiento de más de 500 participantes [120 x 4 (pacientes) + grupos controles + grupo estudio piloto) no parecen ser viables. Además, la falta de una partida presupuestaría dedicada al pago de los participantes, que en el caso de los participantes controles ayudaría a garantizar (en parte) la viabilidad del proyecto, parece confirmar estos riesgos. Finalmente, se hace referencia a la compra de únicamente 120 actígrafos (para más de 500 participantes). Considerando el uso intensivo de los aparatos, así como el tiempo reducido de desarrollo del proyecto, el número de aparatos considerados no parece ser adecuado.

D (Aceptable)

2. Equipo de investigación

El grupo de investigación y de trabajo lo conforman profesionales con perfiles diversos, por lo que el Dr. Rivera-Romero ha conseguido conformar un grupo altamente interdisciplinar y competente en las áreas relevantes del proyecto. Además, diferentes miembros del grupo de investigación y trabajo han demostrado una buena sinergia y capacidad de trabajo, si se consideran las publicaciones conjuntas, y la colaboración activa en el marco del proyecto "Understanding Daily Multiple Sclerosis related Fatigue: a Participatory Health Informatics Approach (MSF-PHIA, 01-02-2020- 30-04-2022)". El CV del Dr. Rivera-Romero muestra una carrera investigadora consolidada en las áreas de Tecnologías aplicadas a la salud y Tecnologías asistenciales, lo que sugiere un perfil investigador interdisciplinar y adecuado para la propuesta presentada. Sin embargo, aunque el Dr. Rivera-Romero haya participado en un número relevante de proyectos de investigación, obtenidos en convocatorias competitivas de índole nacional e internacional, no acredita méritos suficientes para liderar la propuesta presentada. Su producción científica, así como su aparente contribución a la misma si se considera la posición de firma, es modesta y su experiencia en liderazgo parece insuficiente para dirigir un proyecto de esta envergadura.

C (Bueno)

3. Impacto

3.1. Impacto científico-técnico

Aunque el plan de comunicación de las aportaciones científico-técnicas y el plan de divulgación de los resultados son adecuados, no queda sufrientemente claro la diferencia entre los avances científicos del proyecto MyFatigue comparado con los del proyecto MSF-PHIA. Ambos proyectos están basados en inteligencia artificial interpretativa y explicable para identificar tipologías de pacientes en base a su fatiga crónica.

C (Bueno)

3.2. Impacto social y económico

En la misma línea, los resultados de MyFatigue puede que tengan un impacto social y económico sólo incremental respecto al proyecto MSF-PHIA, ya que ambos intentan generar conocimiento y modelos valiosos para la personalización de intervenciones de manejo de la fatiga crónica en poblaciones de pacientes similares. Además, no se especifica de forma clara cómo los resultados derivados del proyecto podrían tener un impacto positivo en la calidad de la vida de los pacientes (y cómo se mediría/cuantificaría dicho impacto).

C (Bueno)

Valoración Global

C, Bueno

PARTE 2

OTROS ASPECTOS A CONSIDERAR

a) Capacidad formativa (cumplimentar solo cuando se haya solicitado la inclusión del proyecto en la convocatoria de contratos predoctorales para la formación de doctores). En caso de No aplicar, indicar No aplica en el desplegable y en la caja de texto.

No procede

No aplica

b) Aspectos relacionados con zonas polares o campañas oceanográficas (cumplimentar solo en los proyectos que proceda)

No procede

c) Condiciones específicas para la ejecución de determinados proyectos (cumplimentar solo en los proyectos con aspectos relacionados con las condiciones o implicaciones recogidas en el Anexo IV de la convocatoria).

En los apartados 4.5 y 7, se describe la estrategia para la protección y seguridad de los datos (altamente sensibles) recogidos. De todas formas, no queda claro si la adaptación de la MyFatigue-PRO App (versión modificada de la MSF-PHIA App) sigue la nuevas directrices específicas para apps para monitorización de la actividad física y el bienestar y la salud en dispositivos móviles (Agencia Española de Protección de Datos, 2019).
