# Supplementary material for: Personalized Management of Fatigue in Individuals With Myalgic Encephalomyelitis/Chronic Fatigue Syndrome and Long COVID Using a Smart Digital mHealth Solution: Protocol for a Participatory Design Approach
Source: JMIR Res Protoc. 2024 Apr 12;13:e50157. doi: 10.2196/50157 (PMC11053387; doi:10.2196/50157)
Supplement: Multimedia Appendix 2 [file resprot_v13i1e50157_app2.pdf]

DeepL

Subscribe to DeepL Pro to translate larger documents.
Visit www.DeepL.com/pro for more information.

MINISTERIO
DE CIENCIA
E INNOVACIÓN

AGENCIA
ESTATAL DE
INVESTIGACIÓN

Knowledge Generation Projects 2021<br>Modality: Type B Oriented Research	
**Reference:**	PID2021-125528OB-I00
**Area:**	Information and communication technologies
**Subarea:**	Computer science and information technology
**Principal Investigator**	RIVERA ROMERO, OCTAVIO
**Title:**	PERSONALIZED JUST-IN-TIME FATIGUE MANAGEMENT USING A CONTEXT-AWARE INTELLIGENT DIGITAL SOLUTION: PATIENT-CENTERED PARTICIPATORY APPROACH

KNOWLEDGE GENERATION PROJECTS 2021. MODALITY: TYPE B ORIENTED RESEARCH

SCIENTIFIC AND TECHNICAL EVALUATION REPORT - TECHNICAL COMMISSION

PART 1

EVALUATION CRITERIA

1. Quality and feasibility of the

proposal UMBRAL 30

Score from 0 to 40: 36.0

1.1. Quality

This project presents a solid and consistent proposal. The proposal is well justified and its relevance is clear. The contribution of the proposal to the generation of knowledge in the area of the proposal is well argued. The objectives are clearly and precisely defined.

Score 0 to 30: 27.0

1.2 Feasibility

Feasibility is well demonstrated. The proposed activities are adequate to achieve the proposed objectives. The distribution of tasks among the members of the research team is adequate.

Score 0 to 10: 9.0

2. UMBRAL 20 research team

The research team has the appropriate experience to achieve the objectives of the project. The team members demonstrate their ability to develop the project.

Score 0 to 30: 25.0

3. Impact

THRESHOLD 10

Score from 0 to 30: 27.0

3.1. Scientific and technical impact of expected results.

The project promises to achieve important knowledge advances and significant contributions to the solution of needs of the selected thematic priority. The scientific communication and dissemination plan is well defined.

Score 0 to 20: 17.5

3.2. Social and economic impact of the results

The results of the project have a very clear social impact. The collaboration with companies that will contribute to the project to achieve the socio-economic impact is positively valued. Taking into account the above considerations, the competitive nature of the call and the available budget, it has not been possible to grant all the funding requested.

Score 0 to 10: 9.5

Overall Rating

Score 0 to 100: 88.0

PART 2

OTHER ASPECTS TO CONSIDER

a) Aspects related to polar areas or oceanographic campaigns (Complete only in projects where applicable).

Not applicable

b) Specific conditions for the execution of certain projects (To be completed only for projects with aspects related to the conditions or implications listed in Annex IV of the call for proposals).

Not applicable

EXPERT-REPORT 1

PART 1

EVALUATION CRITERIA

1. Quality and feasibility of the proposal

1.1 Quality

The proposal is clearly oriented to the selected thematic priority: 1.- Health. It intends to study the impact of the combination of different factors on severe fatigue in myalgic encephalomyelitis/chronic fatigue syndrome (ME/CFS) and persistent COVID, as well as the similarity and differences with the use of Artificial Intelligence (AI) tools, which is a need of the selected thematic priority. The proposal adequately justifies this need and its relevance to the well-being of patients with severe fatigue. The starting hypothesis is well grounded, having similarities with previous studies, which detracts from the originality of the proposal. The specific objectives are clearly presented, taking into account from the study of the current state of the art to the analysis of the benefits of the final product. They are in accordance with the work team and the established duration. However, the objectives related to the scientific and technological advancement of the AI tools to be used are limited. The multidisciplinary character is broad with participants from different fields, the predominant ones being computer science and medicine. However, they are not sufficiently balanced with respect to the tasks scheduled.

B (Very Good)

1.2 Feasibility

Different activities are defined to achieve all the proposed objectives; however, it is not sufficiently justified that they are sufficient for the purpose pursued, since it can be seen that patients have to fill in a large number of questionnaires, which shows a significant risk. The number of variables to be considered automatically is limited. Finding enough patients to obtain the initial database, as well as for validation is a complicated task, which is rightly taken into account in the proposal as a risk, although the mitigation plan is insufficient to justify compliance, mainly due to the commitment to be made by the patients. The previous experience and current developments of the team support the achievement of the tasks as planned. The distribution in tasks is adequate and they have the necessary resources to carry out the activities. The proposal does not adequately justify the amount allocated to personnel, nor the time they would have to be hired for.

C (Good)

2. Research team

The PI has sufficient previous experience to lead the proposal, although he does not have extensive experience in project leadership tasks. The research team, in general, has an adequate trajectory to develop the entrusted tasks, although some of the members have decreased their intensity in scientific production in the last years. There is a previous collaboration with foreign researchers, some of whom are included in the Work Team, although there is no evidence of an intensification of these collaborations in the proposal and budget.

C (Good)

3. Impact

3.1. Scientific and technical impact

The final product will be determinant for the detection of severe fatigue, therefore, in this aspect, it will contribute to a thematic priority need. However, its usefulness beyond giving a recommended diagnosis is unclear. In determining representative variables of ME/CFS and persistent COVID fatigues and their differences will be a milestone in the field, however, the achievement of this objective is not sufficiently justified. Furthermore, the expected scientific advances in AI will be limited. The multidisciplinary nature of the proposal will lead to a more robust final product with better results. A standard dissemination plan is presented based on the publication of scientific articles in conferences and journals, with specific indicators, although not very ambitious. The transfer plan is based mainly on the interest of several companies in the results of the project, which is remarkable, although limited. The plan for data processing is very well framed and designed.

D (Acceptable)

3.2. Social and economic impact

The social impact is clear through citizens with intense fatigue problems. The proposal presents an adequate plan for disseminating the results to society, which is essentially based on the edition of a web page, the use of social networks and press releases. It also considers indicators to measure the impact of the programmed activities. The gender dimension is adequately considered. The end users are also taken into account and are a fundamental part of the project, although their massive participation is not guaranteed.

C (Good)

Overall Rating

C=Good

PART 2

OTHER ASPECTS TO CONSIDER

a) Training capacity (to be completed only when the project has been requested to be included in the call for pre-doctoral contracts for the training of doctors). In case of Not applicable, indicate Not applicable in the drop-down and in the text box.

Not applicable.

Not applicable

b) Aspects related to polar areas or oceanographic campaigns (to be completed only for projects where applicable)

Not applicable.

c) Specific conditions for the execution of certain projects (to be completed only for projects with aspects related to the conditions or implications listed in Annex IV of the call for proposals).

Adequately justifies the procedures and protocols it plans to apply.

EXPERT-REPORT 2

PART 1

EVALUATION CRITERIA

1. Quality and feasibility of the proposal

1.1 Quality

The project is in line with the thematic priority of the PEICTI and is framed within the field of health and welfare. The proposal is clear and interesting and attacks a type of problem with a lot of potential. The proposal is relatively novel and well justified. And the starting hypothesis is original and novel. The objectives are clear and realistic and commensurate with the duration of the project. The contribution of the proposal will generate important results in terms of knowledge generation. It is a multidisciplinary proposal with the participation of researchers from different fields.

B (Very Good)

1.2 Feasibility

The proposed activities are in line with the proposed objectives and a correct methodology with an adequate distribution of tasks and identification of critical points is suggested. Previous results in terms of publication of results and participation in projects support the feasibility of the proposal. The team has sufficient resources and potential to achieve the project objectives. The budget presented is reasonable, although with some aspects that are not well justified, such as personnel and payments for open publication.

B (Very Good)

2. Research team

The PI presents a good CV with relevant contributions in different aspects although he has not led national plan projects before, although he has participated in projects relevant to the present proposal. In general it is considered that he has sufficient merits to lead the proposal. The team is clearly multidisciplinary with relevant contributions and a recent history of shared publications. The specialization of its members is adequate and the outsourcing of the proposed research to 2 hospitals in different geographical areas is considered positive. There is a high degree of internationalization with interesting relationships with relevant groups and researchers. The participation and distribution of the team in the objectives and tasks of the project is adequate.

B (Very Good)

3. Impact

3.1. Scientific and technical impact

The proposal has a high degree of novelty and therefore of risk when assessing the scientific impact of the possible results. Even so, it is considered interesting in this aspect. A significant advance of knowledge in the different fields is foreseen. Although in the proposal a greater detail is appreciated in the data and in the definition of the problems from the medical point of view in relation to the technological-scientific problems. The results communication plan is adequate and a plan for internationalization and valorization of results through relations with companies is proposed. The management of data generated by the project is adequate.

B (Very Good)

3.2. Social and economic impact

The proposal implies a clear social impact given the object of study. The results dissemination plan is adequate and there is a gender dimension naturally associated with the pathologies considered. In general, a clear benefit for society is perceived. The project adequately considers the participation of end users and this is adequately managed.

Overall Rating

A=Exceptional

PART 2

OTHER ASPECTS TO CONSIDER

a) Training capacity (complete only when the project has been requested to be included in the call for pre-doctoral contracts for the training of doctors). In case of Not applicable, indicate Not applicable in the drop-down and in the text box.

-

Not applicable

b) Aspects related to polar areas or oceanographic campaigns (to be completed only in projects where applicable)

-

c) Specific conditions for the execution of certain projects (to be completed only for projects with aspects related to the conditions or implications listed in Annex IV of the call for proposals).

-

EXPERT-REPORT 3

PART 1

EVALUATION CRITERIA

1. Quality and feasibility of the proposal

1.1 Quality

The proposal presented in this MyFatigue project is the #Personalized just-in-time management of fatigue using a context-aware intelligent digital solution: Patient-centered participatory approach# and fits perfectly into the PEICTI thematic priority 1. Health in the thematic area ICT # INF and BME # DPT Diagnostic, prognostic and therapeutic tools. The proposal is novel and in terms of application to this symptom that appears associated with post-viral conditions, such as Myalgic Encephalomyelitis/Chronic Fatigue Syndrome (ME/CFS) and persistent COVID. This fatigue as detailed in the presentation is of great relevance given the impact on the patient's health. The objectives are very well defined and both clinical and technical experts are involved to ensure the success of the project (computer scientists, doctors, psychologists). The contribution of this project can be very relevant given the large number of people affected by the pandemic. The project is ambitious but the previous experience of the researchers and the inclusion in the team of other centers with previous experience and patient associations ensures its good development. For all these reasons, I believe that the project can be considered exceptional.

A (Exceptional)

1.2 Feasibility

The final product Myfatigue is a medical software that uses a hardware (wristwatch activimeter) that is a medical device on the market (must be CE marked as a medical device) and conventional mobiles / tablets / computers to provide information that is used to make decisions for therapeutic purposes. This medical software resulting from the project has the consideration of medical device according to regulation (EU) 2017/745 (MDR) and is classified as class IIa according to rule 11 of Annex VIII. Therefore, it requires the intervention of a Notified Body for its conformity assessment and in order to transfer the result of this project to an end customer and make it viable we should contemplate the regulatory requirements. It does not seem to be contemplated to follow harmonized standards for software development such as EN 62304 and EN 82304 and those associated with usability testing EN 62366, risk management EN 14971 + EN 80002-1, the user manual EN ISO 201417, the quality system EN ISO 13485 and the one applicable to clinical research EN 14155, compiling all these in a technical documentation following annex II and III of the
without whose evidence of application the final result cannot be transferred to a company to obtain the CE marking according to MDR required for its commercialization. Including the purchase of Actigraph GT3X activimeters that are FDA cleared (k181077) for use in the US but do not have the CE marking, mandatory for use in Europe, with intervention of Notified Body (they indicate that they are class I without measurement function # giving instead heart rate measurements), it is recommended to use as it is used to measure a product with CE marking with ON (e. g. Respironics Actiwatch 2).g. Respironics Actiwatch 2) It seems that the regulatory aspects of this project have not been considered or have not been incorporated explicitly in the application therefore understanding that these requirements can be incorporated without problems by the team I value the project as A.

A (Exceptional)

2. Research team

PI1 has never been PI but has experience and leadership in other projects and the participation of other relevant researchers that ensure the viability of this project. The CV of the project members is impressive in relation to the subject matter and proposed development so its success is assured. The project itself is based on the application of previous experience in the MSF-PHIA app project so it is well elaborated and justified in its development and scientific publications. For all these reasons I believe that the project can be valued as exceptional.

A (Exceptional)

3. Impact

3.1. Scientific and technical impact

The proposal is novel and in terms of application to this symptom that appears associated with post-viral conditions, such as Myalgic Encephalomyelitis/Chronic Fatigue Syndrome (ME/CFS) and persistent COVID. This fatigue, as detailed in the presentation, is of great relevance given the impact on the patient's health, ensuring that the results will be novel and the subject of publication in scientific journals and congresses. The transfer and valorization plan is not developed as it has possibly not been contemplated as it is

The impact can be assessed as exceptional, however, and I believe that the impact can be assessed as exceptional. However, I believe that the impact can be assessed as exceptional.

A (Exceptional)

3.2. Social and economic impact

The dissemination/dissemination plan is highly developed from the scientific point of view of the different collaborators and PIs, the methodology contemplates the treatment of gender and disability adequately. The results include a medical software incorporating AI that must be elaborated within a quality system and gathering all the regulatory requirements in order to be directly applicable to its later exploitation. Even in the case of thinking in a non-commercializable software but for the own use of the health center (in-house) this is contemplated in the new MDR regulation and the regulatory requirements of compliance with RGSF of Annex I are demanded, thus applying all the indicated regulations. The interest of six companies in the possible results of the project demonstrates the relevance of this project, which will undoubtedly be transferred to the market. I believe that the impact can be considered as exceptional

A (Exceptional)

Overall Rating

A, Exceptional

PART 2

OTHER ASPECTS TO CONSIDER

a) Training capacity (to be completed only when the project has been requested to be included in the call for pre-doctoral contracts for the training of doctors). In case of Not applicable, indicate Not applicable in the drop-down and in the text box.

NA

Not applicable

b) Aspects related to polar areas or oceanographic campaigns (to be completed only for projects where applicable)

NA

c) Specific conditions for the execution of certain projects (to be completed only for projects with aspects related to the conditions or implications listed in Annex IV of the call for proposals).

With respect to ethical aspects, the project details its application in the pilot study, but it should be incorporated in all the preliminary studies in which patients participate and it is indicated that approval is requested from the IRBs of the health centers, and the use of informed consents seems to establish this. Since MyFatigue is a computer program considered to be a medical device, in addition to the approval of the Ethics Committees, the approval of the AEMPS must also be obtained. According to the new MDR regulation, the application must be submitted to the European database EUDAMED (if it is operational at the time of the trials).

EXPERT-REPORT 4

PART 1

EVALUATION CRITERIA

1. Quality and feasibility of the proposal

1.1 Quality

The proposal presented is exhaustive, but, at times, the exposition is superficial. That is, some aspects and/or sections have not been sufficiently developed, specified and/or justified. Examples of this lack of concreteness would be: (1) two groups of patients (plus control groups) are established, but neither their main characteristics nor the inclusion and exclusion criteria are described in detail (Task 1.2, Task 3.1); (2) the proposed methodology for data curation (Tasks 2.1, 2.2, SO5), based mainly on identifying clusters, is compromised by a simplistic assumption, which disregards some contextual factors. That is, the proposal assumes that the physical dimension of the "fatigue" construct, based on the information (subjective and motor activity) provided by a patient, is independent of the type of activity during the 14 days, and working in construction would be equivalent to working in an office. Moreover, always considering the methodological aspect, a 14-day longitudinal study is described (Task 1.5), but the statistical approach obviates the temporal relationship between the variables. On the other hand, it is not clear whether the 14 days of data collection will include the adaptation days (usually 3) that are usually discarded when working with actigraphy. Finally, the decision to position the actigraph on the dominant wrist (instead of the most common and recommended solution: non-dominant wrist) or the collection of other psychophysiological variables (heart rate, etc.) are not clearly justified. The research hypotheses presented are very generic and hardly refutable. Given the experience gained/derived from the project "Understanding Daily Multiple Sclerosis related Fatigue: a Participatory Health Informatics Approach", in which the same PI and some members of the research and working groups participate, it would not be justifiable to claim that it is because the project is exploratory in nature. Moreover, the use of preliminary searches (section 2.3) to motivate the importance of the project are not justified in a team with experience in the subject. Such searches use clearly biased search equations (language, database, or the inclusion of arbitrary search limiters) to provide poor results and do not present a realistic and complete state of the art.

C (Good)

1.2 Feasibility

Although the research team and work team reflect the multi/interdisciplinary nature of the proposal, the project follows an overly techno-centric approach. For example, reference is made to user-based design (UCD, task 2.4), but the MyFatigue prototype will be evaluated by usability experts (rather than users, who are the patients). In addition, the development of the system does not follow an iterative design-test-redesign process typical of UCD. Thus, the organization of the work packages would not guarantee the correct development of the system. In other words, the results of WP3 (testing), starting only in the last months of the project, would not feed back to WP2. Finally, the team lacks experts in human factors and user experience who could properly lead this task. The presence of experts in psychology or psychological intervention, which should be the most relevant part of CATCH, is anecdotal compared to the other profiles. On the other hand, it is not clear whether the MyFatigue App-PRO has been developed for Android or IOS environments (or both). In the case that the App was compatible only with a specific environment, a contingency strategy (e.g., providing a mobile device compatible with the App) is not presented. Considering the lifetime of the project and the budget considered, the development of 7 specific objectives as well as the recruitment of more than 500 participants [120 x 4 (patients) + control groups + pilot study group) do not seem to be feasible. In addition, the lack of a budget line dedicated to the payment of participants, which in the case of control participants would help to (partly) guarantee the feasibility of the project, seems to confirm these risks. Lastly, reference is made to the purchase of only 120 pens (for more than 500 participants). Considering the intensive use of the devices, as well as the reduced development time of the project, the number of devices considered does not seem to be adequate.

D (Acceptable)

2. Research team

The research and working group is made up of professionals with diverse profiles, so that Dr. Rivera-Romero has managed to form a highly interdisciplinary and competent group in the relevant areas of the project. In addition, different members of the research and work group have demonstrated a good synergy and working capacity, considering the joint publications, and the active collaboration in the framework of the project "Understanding Daily Multiple Sclerosis related Fatigue: a Participatory Health Informatics Approach (MSF-PHIA, 01-02-2020- 30-04-2022)". Dr. Rivera-Romero's CV shows a consolidated research career in the areas of Applied Health Technologies and Assistive Technologies, which suggests an interdisciplinary research profile suitable for the proposal submitted. However, although Dr. Rivera-Romero has participated in a relevant number of research projects, obtained in national and international competitive calls, he does not have sufficient merits to lead the proposal presented. His scientific production, as well as his apparent contribution to it considering his signature position, is modest and his leadership experience seems insufficient to lead a project of this magnitude.

C (Good)

3. Impact

3.1. Scientific and technical impact

Although the communication plan of the scientific-technical contributions and the dissemination plan of the results are adequate, the difference between the scientific advances of the MyFatigue project compared to those of the MSF-PHIA project is not sufficiently clear. Both projects are based on interpretive and explainable artificial intelligence to identify typologies of patients based on their chronic fatigue.

C (Good)

3.2. Social and economic impact

Along the same lines, the results of MyFatigue may have only an incremental social and economic impact with respect to the MSF-PHIA project, as both attempt to generate valuable knowledge and models for the personalization of chronic fatigue management interventions in similar patient populations. In addition, it is not clearly specified how the results derived from the project could have a positive impact on patients' quality of life (and how such an impact would be measured/quantified).

C (Good)

Overall Rating

C, Good

PART 2

OTHER ASPECTS TO CONSIDER

a) Training capacity (to be completed only when the project has been requested to be included in the call for pre-doctoral contracts for the training of doctors). In case of Not applicable, indicate Not applicable in the drop-down and in the text box.

Not applicable

Not applicable

b) Aspects related to polar areas or oceanographic campaigns (to be completed only for projects where applicable)

Not applicable

c) Specific conditions for the execution of certain projects (to be completed only for projects with aspects related to the conditions or implications listed in Annex IV of the call for proposals).

In sections 4.5 and 7, the strategy for the protection and security of the (highly sensitive) data collected is described. Anyway, it is not clear whether the adaptation of the MyFatigue-PRO App (modified version of the MSF-PHIA App) follows the new specific guidelines for apps for monitoring physical activity and well-being and health on mobile devices (Agencia Española de Protección de Datos, 2019).
